# Improved Oocyte Isolation and Embryonic Development of Outbred Deer Mice

**DOI:** 10.1038/srep12232

**Published:** 2015-07-17

**Authors:** Jung Kyu Choi, Xiaoming He

**Affiliations:** 1 Department of Biomedical Engineering, The Ohio State University, Columbus, OH 43210.; 2 Davis Heart and Lung Research Institute, The Ohio State University,Columbus, OH 43210.; 3 James Comprehensive Cancer Center, The Ohio State University, Columbus, OH 43210.

## Abstract

In this study, we improved the protocol for isolating cumulus-oocyte complexes (COCs) from the outbred deer mice by using only one hormone (instead of the widely used combination of two hormones) with reduced dose. Moreover, we identified that significantly more metaphase II (MII) oocytes could be obtained by supplementing epidermal growth factor (EGF) and leukemia inhibition factor (LIF) into the previously established medium for *in vitro* maturation (IVM) of the COCs. Furthermore, we overcame the major challenge of two-cell block during embryonic development of deer mice after either *in vitro* fertilization (IVF) or parthenogenetic activation (PA) of the MII oocytes, by culturing the two-cell stage embryos on the feeder layer of inactivated mouse embryonic fibroblasts (MEFs) in the medium of mouse embryonic stem cells. Collectively, this work represents a major step forward in using deer mice as an outbred animal model for biomedical research on reproduction and early embryonic development.

The genus Peromyscus are rodents indigenous to *North America* and contain the species commonly called deer mice because their fur color resembles that of deer^1^,^2^. Due to their outbred nature similar to that of humans, deer mice have been used as an animal model for a variety of studies on phylogeography, speciation, chromosomes, and population genetics and evolution^3^,^4^,^5^,^6^. However, the use of deer mice as an animal model for research on reproduction and early embryonic development has been challenging. This is because less than 5 oocytes per animal could be obtained after superovulation with the widely used hormonal combination of pregnant mare’s serum gonadotropin (PMSG) to stimulate growth and maturation of ovarian follicles and human chorionic gonadotropin (hCG) to induce ovulation of cumulus-oocyte complexes (COCs) from the matured follicles in ovary^1^,^2^,^7^,^8^. In a previous study, we successfully improved the number (~20) of oocytes (including both MII oocytes that are fertilizable and immature ones that are non-fertilizable) per animal isolated from deer mice by *in vitro* maturation (IVM) of cumulus-oocytes complexes (COCs) obtained after injecting the animal with both PMSG and hCG^1^. In addition, *in vitro* fertilization was achieved by combining MII oocytes with sperm, and the fertilized oocytes were successfully developed into 4-cell stage embryos *in vitro*^1^. However, the efficiency of obtaining the 4-cell stage embryos is low, indicating 2-cell block is a significant issue during embryonic development of deer mice. Such two-cell block was also a major challenge for the establishment of an *in vitro* culture system of early embryos in inbred mice^9^,^10^ and hamsters^11^ in the past.

In this study, we further significantly improved the isolation of MII oocytes from deer mice via IVM of COCs by using only one hormone (PMSG without the use of hCG) and by supplementing the medium for IVM of COCs with epidermal growth factor (EGF) and leukemia inhibition factor (LIF). In addition, we successfully developed a protocol to achieve parthenogenetic activation (PA) of the MII oocytes of deer mice. Moreover, we successfully overcame the major challenge of two-cell block during embryonic development of deer mice by culturing the two-cell stage embryos on the feeder layer of inactivated mouse embryonic fibroblasts (MEFs) in medium for culturing embryonic stem cells (ESCs). Therefore, this study represents a major step forward in using deer mice as an outbred animal model that has more translational value than many inbred mouse strains for biomedical research on reproduction and early embryonic development.

## Results and Discussion

### The number of pups born after mating deer mice is similar to the number of oocytes isolated by superovulation

**Figure 1:**
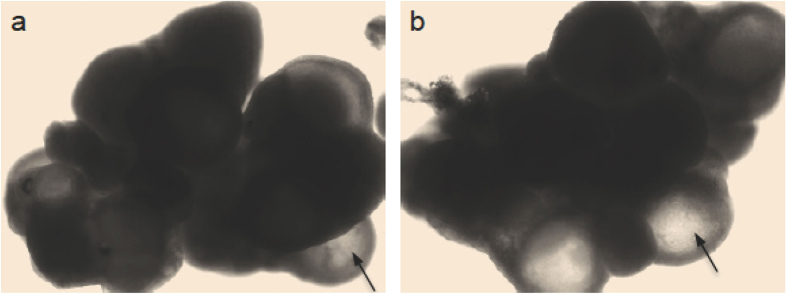
Typical images of ovaries collected from female deer mice following injection with either PMSG only (one dose, **a**) or PMSG & hCG (one PMSG and one hCG at 56 h after PMSG administration, **b**). Arrows indicates antral follicles.

As shown in Table 1, the number of pups born after mating female deer mice with male ones is 4.2 ± 0.4 per female, suggesting that the number of naturally ovulated oocytes should be ~4-5 per female. This quantity is almost the same as the number (4.3 ± 0.5) of oocytes isolated per female by superovulation. In addition, superovulation of genetically identical F1 hybrid laboratory mice usually induces the release of approximately 25–30 oocytes in COCs per animal (e.g., 24 ± 2 per animal for the four female B6CBAF1 mice used in this study). These facts suggest that the commonly used hormonal combination of PMSG and hCG for superovulation may not work for deer mice. To further identify which hormone fails to function, we isolated the ovaries from female deer mice. Typical images of the ovaries from female deer mice injected with either PMSG only or the combination of PMSG and hCG are shown in Fig. 1a,b, respectively. Many antral follicles (arrows) were observable in the ovaries of mice injected with either PMSG only or the combination of PMSG and hCG. Since PMSG is a follicle stimulating hormone (FSH) and functions to facilitate the growth and maturation of immature follicles, the data shown in Fig. 1a,b indicate that PMSG functions as usual in deer mice. On the other hand, hCG is a luteinizing hormone (LH) to induce ovulation of oocyte-containing COCs from the ovary into the oviduct. The data shown in Fig. 1b indicate that hCG fails to induce ovulation of the many antral follicles in the ovary of deer mice.

The low yield of oocytes retrieved by superovulation (or failure of inducing ovulation by hCG) from deer mice could be a result of the varied response of ovarian tissue to exogenous hormones among different rodent species, due to the fact that superovulation does not change the level of endogenous gonadotropins^12^,^13^. Another possible explanation is that the low yield might be caused by stresses that could decrease ovulation in mammals by stimulating the hypothalamus pituitary-gonadal axis to inhibit endogenous gonadotropin secretion^14^,^15^,^16^. Deer mice might be more sensitive to stresses to survive in their environment as an outbred animal. Further studies are needed to identify the exact mechanism that causes the low yield of oocytes following superovulation in deer mice.

### Isolation of COCs from antral follicles and IVM of COCs to obtain MII oocytes of deer mice

**Figure 2:**
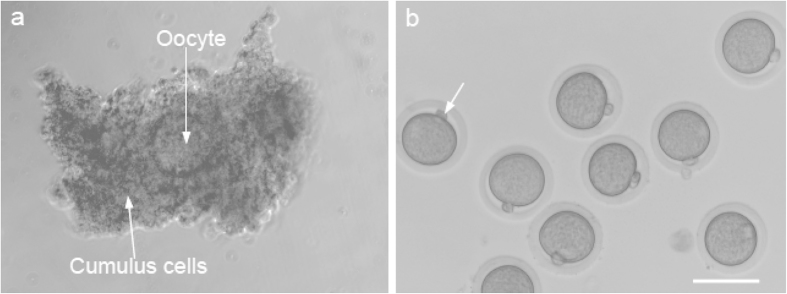
Typical micrographs showing the morphology of cumulus-oocyte complex (COC, **a**) isolated from ovary injected with PMSG only and MII oocytes (**b**) with a first polar body (arrow) isolated from IVM of COCs in culture medium with LIF and EGF. Scale bar: 75 μm.

In view of the fact that hCG fails to induce ovulation of COCs from antral follicles in deer mice ovary, we decided to isolate COCs from the ovaries of deer mice injected with PMSG only although both hormones have been used in the literature^1^,^2^. Two different protocols of PMSG administration have been used for stimulating the growth and maturation of ovarian follicles in deer mice: the 1-PMSG method consisting of one PMSG administration at 5IU per animal followed by isolation of COCs 56 h after the PMSG administration^1^, and the 2-PMSG method that has a second administration of PMSG 24 h after the first one both at 15 IU per animal followed by isolation of COCs 24 h after the second PMSG administration^2^. The COCs were manually retrieved from the ovaries by puncturing the antral follicles using a pair of 30-gauge needles attached to disposable syringes under a stereomicroscope. Fig. 2a presents a typical micrograph showing a single oocyte surrounded by cumulus cells in a COC isolated manually from antral follicle in deer mice administered with PSMG using the 1-PMSG method. The COCs were further matured *in vitro* to obtain MII oocytes using the protocol previously reported^1^. As shown in Table 2, the 1-PMSG method resulted in a higher number of COCs (17.8 ± 4.9) and MII oocytes (11.2 ± 3.1) per mouse than the 2-PMSG approach with 15 ± 4.2 COCs and 7.4 ± 2.7 MII oocytes per mouse, probably because a high dose of PMSG could have an adverse effect on endocrine function and gametogenesis of ovaries^17^,^18^,^19^,^20^ and the cytoskeletal dynamics of oocytes^21^,^22^.

To improve the efficiency of isolating MII oocytes for fertilization and embryonic development studies, we further optimized the previously reported (control^1^) protocol for IVM of COCs. This was done by supplementing the IVM medium with EGF and/or LIF because both EGF and LIF have been shown to play an important role in promoting cytoplasmic and nuclear maturation of oocytes^23^,^24^,^25^,^26^. As shown in Table 3, supplementing the IVM medium with the combination of EGF (5 ng/ml) and LIF (1000 IU/ml) indeed significantly improved the efficiency of developing the immature oocytes in COCs to the MII stage although EGF or LIF alone did not. A typical micrograph showing the typical morphology of MII oocytes (obtained with the improved IVM method) with a polar body (arrow) and shiny cytoplasm semi-enclosed in a zona pellucida is given in Fig. 2b. It is exciting that the efficiency of deriving MII oocytes from the manually collected COCs could be significantly improved to more than 80% with the improved IVM method since only the MII oocytes could be fertilized for further embryonic development.

### Parthenogenetic activation (PA) and *in vitro* fertilization (IVF) of MII oocytes for embryonic development of deer mice

**Figure 3:**
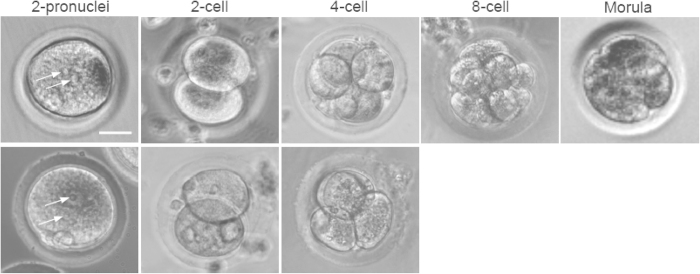
Embryonic development of two-pronuclei embryos obtained by parthenogenetic activation (PA, top row) and *in vitro* fertilization (IVF, bottom row) cultured on the feeder layer of inactivated mouse embryonic fibroblasts (MEFs) in embryonic stem cell (ESC) medium, showing the development to 4-cell stage of the parthenogenetically activated and *in vitro* fertilized embryos, respectively. The parthenogenetically activated oocytes could further develop to 8-cell and morula stages. Arrows indicate pronuclei. Scale bar: 15 μm.

As shown in Table 4, we successfully conducted PA for the first time on MII oocytes from deer mice and the efficiency of successful activation of the oocytes by PA judged by the formation of two pronuclei (arrows in Fig. 3) is similar to the efficiency of fertilizing the MII oocytes by IVF. Moreover, the efficiency of developing to the 2-cell stage was even higher for the parthenogenetically activated oocytes than the *in vitro* fertilized ones.

A major challenge to the embryonic development of embryos from deer mice is the two-cell block and few fertilized oocytes could develop to the 4-cell stage by conventional culture in embryo medium (no MEFs) in Petri dish (Table 4). Actually, this problem of two-cell block during embryonic development *in vitro* may occur to most outbred and inbred strains of mice^27^,^28^, probably due to the fact that the *in vitro* culture condition does not completely recapitulate the native *in vivo* microenvironment of the two-cell embryos^29^. Co-culture with the oviduct and using defined culture medium without glucose and phosphate have been reported to overcome two-cell block in early embryos of several species^30^,^31^,^32^.

In this study, we introduced a co-culture system by cultivating the two-cell stage embryos on mouse embryonic fibroblasts (MEFs) to overcome the two-cell block of deer mouse embryos derived by IVF and PA. As shown in Table 4, this co-culture system significantly improves the development of the two-cell stage embryos to the 4-cell stage when the defined mouse embryonic stem cell (ESC) culture medium is used. Moreover, embryos derived from PA further developed to the 8-cell and morula stage. Typical images of the embryos from PA and IVF at the various stages cultured on the MEF feeder layer in ESC medium are shown in Fig. 3. Interestingly, no 2-cell embryos from PA cultured on the MEF feeder layer in embryo culture medium developed to the 4-cell stage although it is not statistically different from the culture condition of embryo medium with no MEF feeder layer. Further research is needed to identify the exact mechanism or the specific factors in the co-culture system with mouse ESC medium helps to overcome the two-cell block and to further develop the embryos to the stage of implantable blastocysts. As shown in Table S1 and Figure S1, MII oocytes from the hybrid B6CBAF1 mice could be successfully fertilized *in vitro* and further developed to blastocysts, which is the result of several decades of effort from many researchers to optimize the culture condition for embryonic development of the laboratory mice^33^. The embryo research on deer mice is in the early stage and successful IVF of MII oocytes from deer mice and development of the fertilized oocytes to the 2-cell and 4-cell (albeit few) stage was achieved only recently^1^.

In summary, we developed an efficient protocol for isolating MII oocytes from deer mice using only one hormone (PMSG) at a reduced dose (5 IU per animal) and by supplementing EGF and LIF into the medium for IVM of COCs. Moreover, we successfully conducted PA of MII oocytes from deer mice for further embryonic development. Furthermore, we successfully established a co-culture system to overcome the 2-cell block during embryonic development of deer mice and developed the embryos to the morula stage for the first time. This work represents a major step toward the use of deer mice as an outbred animal model that has more translational value than many inbred mouse species for biomedical research on reproduction and early embryonic development. However, more studies are needed to further optimize the culture condition of deer mouse embryos so that they could develop into blastocysts that can be implanted *in vivo* to produce viable offspring.

## Materials and Methods

### Animals

Peromyscus maniculatus bairdii (BW stock) deer mice were purchased from the Peromyscus Genetic Stock Center at the University of South Carolina, Columbia, SC. The inbred B6CBAF1 laboratory mice were purchased from The Jackson Laboratory. All mice were maintained and bred (for deer mice only) on a 16:8 h light-dark cycle. All animal use procedures were approved by the Institutional Animal Care and Use Committee (IACUC) at The Ohio State University (IACUC #: 2011A00000084) and all animal studies were carried out in accordance with the approved guidelines.

### Chemicals

L-15 Leibovitz-glutamax, leukemia inhibitory factor (LIF), and fetal bovine serum (FBS) were purchased from Invitrogen, Chemicon, and Hyclone, respectively. Unless specifically noted otherwise, all other chemicals were purchased from Sigma.

### Breeding for delivery of pups of deer mice

Female deer mice 12 to 14 weeks of age were used for breeding. They were mated with male deer mice of the same age overnight (~13 h) and successful mating was confirmed by the presence of vaginal plug (made of coagulated secretions from the coagulating and vesicular glands of the male) in female deer mice the next morning at ~7 am. To check vaginal plug, the female mice were lifted up by holding the distal end of their tail for better and deeper observation into the vagina. After 21–23 days, the pregnant female deer mice delivered pups and the number of pups was recorded.

### Isolation of oocytes by superovulation from deer and B6CBAF1 mice

For superovulation of deer (12 to 14-week old) and B6CBAF1 (6 to 8-week old) mice, PMSG (5IU) was injected (i.p.) into the female mice and hCG at the same dose was applied 56 h later. Oocytes were isolated from the oviduct 15 h after hCG administration. Superovulated cumulus–oocyte complexes (COCs) were retrieved by flushing the oviducts with M2 medium. To remove cumulus cells, the COCs were incubated in M2 medium containing 200 IU/ml hyaluronidase at 37 °C for up to 3 min and further washed twice using fresh M2 medium to obtain clean oocytes.

### Isolation of COCs from antral follicles of deer mice

The ovaries retrieved from euthanized female deer mice after injected with PMSG were placed in L-15 Leibovitz-glutamax medium supplemented with 10% (v/v) heat-inactivated FBS. COCs were isolated from antral follicles in the ovaries mechanically by carefully and gently puncturing the follicles using a pair of 30-gauge needles attached to disposable syringes under a stereomicroscope. The COCs containing immature oocytes in the antral follicles from both the outer (ovarian surface) and inner (deep ovarian tissue) layer of ovarian cortex were collected^1^.

### *In vitro* maturation (IVM) of the COCs of deer mice

To develop optimized IVM methods, COCs were cultured for 19 h in the previously established control medium^1^ supplemented with epidermal growth factor (EGF, 5 ng/ml), LIF leukemia inhibitory factor (LIF, 1000 IU/ml), or the combination of EGF (5 ng/ml) and LIF (1000 IU/ml). The control medium consisted of the minimum essential medium-α containing Earle’s salts (Invitrogen), 10 mg/ml streptomycin sulfate, 75 mg/ml penicillin G, and 5% (v/v) heating activated FBS covered with 250 μl of mineral oil in a 4-well plate at 37 °C in 5% CO_2_ air. After IVM, COCs were collected and incubated in M2 medium containing 200 IU/ml hyaluronidase at 37 °C for up to 3 min to remove cumulus cells, followed by washing twice in fresh M2 medium to obtain clean oocytes. Matured oocytes at the metaphase II stage were determined by the appearance of the first polar body.

### Preparation of mouse embryonic fibroblasts (MEFs)

MEFs were isolated by following a protocol detailed elsewhere^34^. In brief, E13.5 mouse embryos were dissected and the brain, limbs, and internal organs removed to ensure a pure fibroblastic population. Embryos were minced with a sterile razor blade into small pieces and they were incubated at 37 °C in 5% CO_2_ air for 10 min. The samples were then pipetted up and down using a 1 ml pipette to further break up the tissue, followed by culturing in a 100 mm culture dish coated with 0.1% gelatin at 37 °C in 5% CO_2_ air to obtain the MEFs.

### Parthenogenetic activation (PA) and *in vitro* fertilization (IVF) of deer mouse oocytes

For PA, MII oocytes of deer mice were cultured in Ca^2+^-free KSOM medium supplemented with 10 mM SrCl_2_ and 5 μg/ml cytochalasin B for 3.5 h. For IVF, 5 MII oocytes were combined with 2 × 10^4^ sperm in a 200 μl droplet of TYH medium for 4.5 h. To obtain sperm for IVF, 12 to 14-week old male deer mice were euthanized by cervical dislocation and epididymides were collected by dissection. They were then placed in the central well of an IVF dish with 1 ml TYH medium, a modified Krebs-Ringer bicarbonate solution containing 5.56 mM glucose, 1.0 mM sodium pyruvate, 4 mg/ml bovine serum albumin (BSA), and antibiotics. After making 5–7 longitudinal cuts using a syringe needle on each epididymis, they were incubated for 20 min at 37 °C in 5% CO_2_ air to allow for sperm dispersion. The sperm suspensions were then further incubated for 2 h at 37 °C in 5% CO_2_ air for capacitation.

### Embryo culture for deer mice

The parthenogenetically activated and *in vitro* fertilized oocytes were subsequently cultured in a drop of 50 μl of Hoppe and Pitts embryo culture medium^35^ modified by removing sodium lactate and increasing the sodium chloride concentration to 5.97 g/ml at 37 °C in 5% CO_2_ air^1^. Development of the parthenogenetically activated and *in vitro* fertilized oocytes was monitored under a phase contrast light microscope (Nikon 80i) for the formation of two-pronuclei and two-cell embryos. The two-cell embryos were further cultured on MEF feeder (cell density: 2 × 10^4^) layer in either ESC or embryo medium in a 96-well plate. The MEF feeder layer was made by inactivating the MEFs at passage 3 with 10 μg/ml mitomycin. The embryonic stem cell (ESC) medium was made of knockout DMEM supplemented with 15% knockout serum replacement (Invitrogen), 0.1 mM 2-mercaptoethanol (βME, Gibco), 2 mM L-glutamine, antibiotics (1% penicillin/streptomycin), and 1000 U ml^−1^ leukemia inhibition factor (LIF, Millipore). Two-cell embryos were also cultured on petri dish without MEF feeder layer in Hoppe and Pitts embryo medium as control.

### Statistical analysis

A generalized linear model (PROC-GLM) and one-way ANOVA in a Statistical Analysis System (SAS) program was employed for statistical analysis to determine the *p* value between various conditions. The differences were taken as significant when the *p* value was less than 0.05.

## Additional Information

**How to cite this article**: Choi, J. K. and He, X. Improved Oocyte Isolation and Embryonic Development of Outbred Deer Mice. *Sci. Rep.* **5**, 12232; doi: 10.1038/srep12232 (2015).

## Supplementary Material

Supplementary Information

## Figures and Tables

**Table 1:** The correlation between the number of pups born after mating female deer mice with male deer mice and oocytes isolated after superovulating female deer mice.

**No. of mated female mice^a^**	**No. of pups in total (Mean ± SD)**	**No. of superovulated female mice^b^**	**No. of isolated oocytes in total (mean ± SD)**
5	21 (4.2 ± 0.4)	3	13 (4.3 ± 0.5)

^a^Female mice were mated with male mice and the number of pups was checked after birth.

^b^PMSG (5IU) was injected into female mice and hCG at the same dose was applied 56 h later. Oocytes were isolated from the oviduct 15 h after hCG administration.

**Table 2:** A comparison of oocytes retrieved from deer mice after injecting with PMSG using two different methods: GVBD, germinal vesicle breakdown.

**Method**	**Dose (IU)**	**No. of mice treated**	**No. of COCs in total (per animal)**	**No. (%^c^) of oocytes at**		**No. (mean ± SD) of MII oocytes retrieved in total (per animal)**
				**GVBD**	**MII**	
1-PMSG^a^	5	5	89 (17.8 ± 4.9)	89 (100)	56 (63)	56 (11.2 ± 3.1)
2-PMSG^b^	15	5	75 (15 ± 4.2)	75 (100)	37 (49)	37 (7.4 ± 2.7)

^a^The 1-PMSG method consists of one PMSG injections and COCs were isolated 56 h after the PMSG administration.

^b^The 2-PMSG method includes a second administration of PMSG 24 h after the first one at the same dose, and COCs were isolated from ovaries 24 h after the 2^nd^ PMSG administration.

^c^Percentage of the total number of COCs

**Table 3:** The effect of epidermal growth factor (EGF) and leukemia inhibitory factor (LIF) in culture medium on *in vitro* maturation (IVM) of COCs isolated from deer mice: GVBD, germinal vesicle breakdown.

**Group**	**Total No. of COC**	**No. (%^b^) of oocytes at**	
		**GVBD**	**MII**
C^a^	39	39	23 (59^c^)
C+EGF	36	36	20 (56^c^)
C+LIF	36	36	22 (61^c^)
C+EGF+LIF	42	42	35 (83^c^)

^a^C represents control condition, for which COCs were cultured in medium with no EGF or LIF.

^b^Percentage of COCs in total

^c^Different superscripts indicate the difference is significant (*p *< 0.05). In other words, the only significant difference is between C+EGF+LIF and the other groups.

**Table 4:** Embryonic development using different methods of culturing 2-cell embryos obtained by parthenogenetic activation (PA) and *in vitro* fertilization (IVF) of MII oocytes from deer mice. ESC, embryonic stem cell; MEFs: mouse embryonic fibroblasts.

**Type**	**No. of MII oocytes**	**% of 2-pronuclei embryos**	**No. (%^c^) of 2-cell embryos**		**Culture method**	**No. (%^d^) of embryos developed to**		
						**4-cell**	**8-cell**	**Morula**
PA	269	88^a^	121 (44)	32	Embryo medium, no MEFs	4 (12^e^)	0	0
				34	Embryo medium on MEFs	0^e^	0	0
				55	ESC medium on MEFs	30 (54^e^)	8 (14)	2 (3)
IVF	200	87^b^	66 (33)	30	Embryo medium, no MEFs	8 (26)	0	0
				36	ESC medium on MEFs	15 (41)	0	0

^a^A total of 93 fertilized oocytes were monitored for the formation of two pronuclei.

^b^A total of 80 fertilized oocytes were monitored for the formation of two pronuclei.

^c^Percentage of MII oocytes

^d^Percentage of the total number of 2-cell embryos used for the specific culture condition

^e^Different superscripts indicate the difference is significant (*p *< 0.05). In other words, the only significant difference is between the group of ESC medium on MEFs and the other groups for parthenogenetic activation.
