# Supplementary material for: Improved Oocyte Isolation and Embryonic Development of Outbred Deer Mice
Source: Sci Rep. 2015 Jul 17;5:12232. doi: 10.1038/srep12232 (PMC4648404; doi:10.1038/srep12232)
Supplement: Supplementary Information [file srep12232-s1.pdf]

Supplementary Information

Improved Oocyte Isolation and Embryonic Development of Outbred Deer Mice

Jung Kyu Choi[1,2] and Xiaoming He[1,2,3]*

[1]Department of Biomedical Engineering, [2]Davis Heart and Lung Research Institute, and [3]James Comprehensive Cancer Center, The Ohio State University, Columbus, OH 43210

* Correspondence should be addressed to:
Xiaoming He, Ph.D.
Department of Biomedical Engineering
The Ohio State University
1080 Carmack Road
Columbus, OH 43210
Phone: (614) 292-6992
E-mail : He.429@osu.edu

Supplementary Methods

***In vitro* fertilization (IVF) and embryo culture of B6CBAF1 mice.** To obtain sperm for IVF, 6 to 8-week old male B6CBAF1 mice were euthanized by cervical dislocation and epididymides were collected by dissection. They were then placed in the central well of an IVF dish with 1 ml KSOM. After making 5–7 longitudinal cuts using a syringe needle on each epididymis, they were incubated for 20 min at 37 °C in 5% CO_2 air to allow for sperm dispersion. The sperm suspensions were further incubated in KSOM for 2 h at 37 °C in 5% CO_2 air for capacitation. For IVF, 5 MII oocytes of B6CBAF1 were inseminated with 2×10^4 sperm in a 200 µl droplet of KSOM for 4.5 h. Fertilized oocytes were subsequently cultured in a drop of 50 µl of CZB medium at 37 °C in 5% CO_2 air. Development of the oocytes was monitored under a phase contrast light microscope (Nikon 80i) for the formation of 2-pronuclei, 2-cell, 4-cell, morula, and blastocyst at various intervals for up to 5 days.

Table S1. Development of embryos derived from *in vitro* fertilization (IVF) of metaphase II (MII) oocytes of B6CBAF1 mice.

No. of MII oocytes	% of 2-pronuclei embryos	No. (%[b]) of 2-cell embryos	No. (%[c]) of embryos developed to		
			4-cell embryo	Morula	Blastocyst
72	92[a]	45 (63)	36 (80)	27 (60)	21 (47)

[a]A total of 38 fertilized oocytes was monitored for the formation of two pronuclei.

[b]Percentage of MII oocytes

[c]Percentage of 2-cell embryos

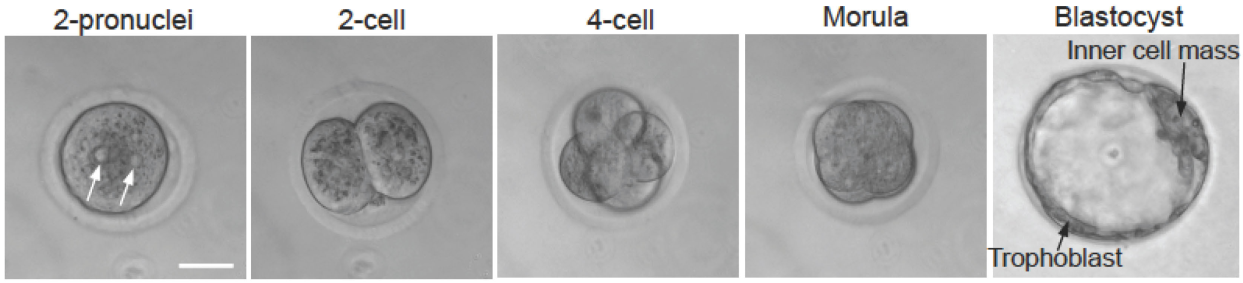

Figure S1. Embryonic development of the inbred B6CBAF1 laboratory mice after *in vitro* fertilization (IVF). White arrows indicate two pronuclei and the fertilized oocytes developed to 2-cell, 4-cell, morula, and blastocyst stage *in vitro*. Scale bar: 30 µm.
